# Surgical Treatment of a Giant Pleomorphic Adenoma of the Submandibular Gland: A Case Report

**DOI:** 10.3389/fsurg.2021.800563

**Published:** 2022-01-25

**Authors:** Zehui Wu, Defeng Liu, Shihao Peng, Yuejun Wang, Xiaolin Zhan, Laibin Li, Hong Wan, Yangyang Li, Tao Guo, Aman Xu

**Affiliations:** ^1^Department of General Surgery, The First Affiliated Hospital of Anhui Medical University, Hefei, China; ^2^Department of General Surgery, The Fourth Affiliated Hospital of Anhui Medical University, Hefei, China; ^3^Department of Pathology, The Fourth Affiliated Hospital of Anhui Medical University, Hefei, China; ^4^Department of Ultrasound, The Fourth Affiliated Hospital of Anhui Medical University, Hefei, China; ^5^Department of Radiology, The Fourth Affiliated Hospital of Anhui Medical University, Hefei, China

**Keywords:** pleomorphic adenoma, salivary gland neoplasm, submandibular gland, diagnosis, treatment, recurrence

## Abstract

Pleomorphic adenomas (PAs) are the most common benign salivary neoplasms. PAs are generally slow-growing but may sometimes become aggressive and grow rapidly within a short period of time. Here, we report the case of an 83-year-old Chinese woman with an anterior neck mass that had been growing over the past 30 years. She felt uncomfortable because the mass had grown quite rapidly in the past year. The final diagnosis of a PA of the left submandibular gland was confirmed by histopathological and immunohistochemical examinations after surgical resection. Our patient recalled a history of an excision of a neck mass 40 years prior to presentation at another hospital. Based on our imaging findings and surgical findings, we speculate that the neck mass 40 years prior may also have been a PA. Our case reminds us the rare recurrence possibility of PAs, and early and thorough resection may have a good prognosis. In addition, to the best of our knowledge, this is the largest PA of the submandibular gland reported to date.

## Introduction

Pleomorphic adenomas (PAs) are the most common subtype of benign salivary gland neoplasm, accounting for 70–80% of all benign salivary gland tumors (1). PAs are generally slow-growing but may sometimes become aggressive and grow rapidly within a short period of time. PAs occur most frequently in women between the ages of 40 and 50 years (2). Here, we report the case of an 83-year-old Chinese woman with a large anterior neck mass that had been growing over the past 30 years. Surgical resection confirmed that the PA was derived from the left submandibular gland. Her clinical features and relevant literature are reviewed to discuss the aspects that we need to pay attention to in the diagnosis and treatment of this rare condition.

## Case Presentation

### Chief Complaints

An 83-year-old Chinese woman with a large anterior neck mass that had been growing over the past 30 years was referred to our ward. She complained that the mass had grown so rapidly in the past year that she was unable to breathe easily when lying supine and had to sleep on her side. However, in the standing position, she showed no symptoms of dyspnea and was able to complete the tasks of the day with ease.

### History of Present Illness

Thirty years prior to presentation at our hospital, the patient noticed a 2 cm solid mass located on the left side of her neck. She did not experience any palpation pain, weight loss, fever or chills, dysphagia, hoarseness, or rupture of the mass at the time. The mass had been growing slowly since then but without any discomfort. In 2020, the maximum diameter of the mass reached 10 cm. Since January 2021, the patient noticed that the mass had grown more rapidly, along with swelling and pain. As a result of the rapid growth of the tumor, she now had obvious difficulties breathing when lying supine and had to sleep on her side. However, she still had no dysphagia or hoarseness. She was suspected of having a thyroid tumor by the local clinic and was recommended to our hospital for further treatment.

### History of Past Illness

It is noteworthy that the patient underwent an excision of a neck mass 40 years prior to presentation at another hospital. However, specific data could not be retrieved, and the pathological diagnosis at that time was not known. She has no other illnesses besides this.

### Family History

She had no family history of similar illnesses.

### Physical Examination Upon Admission

**Figure 1:**
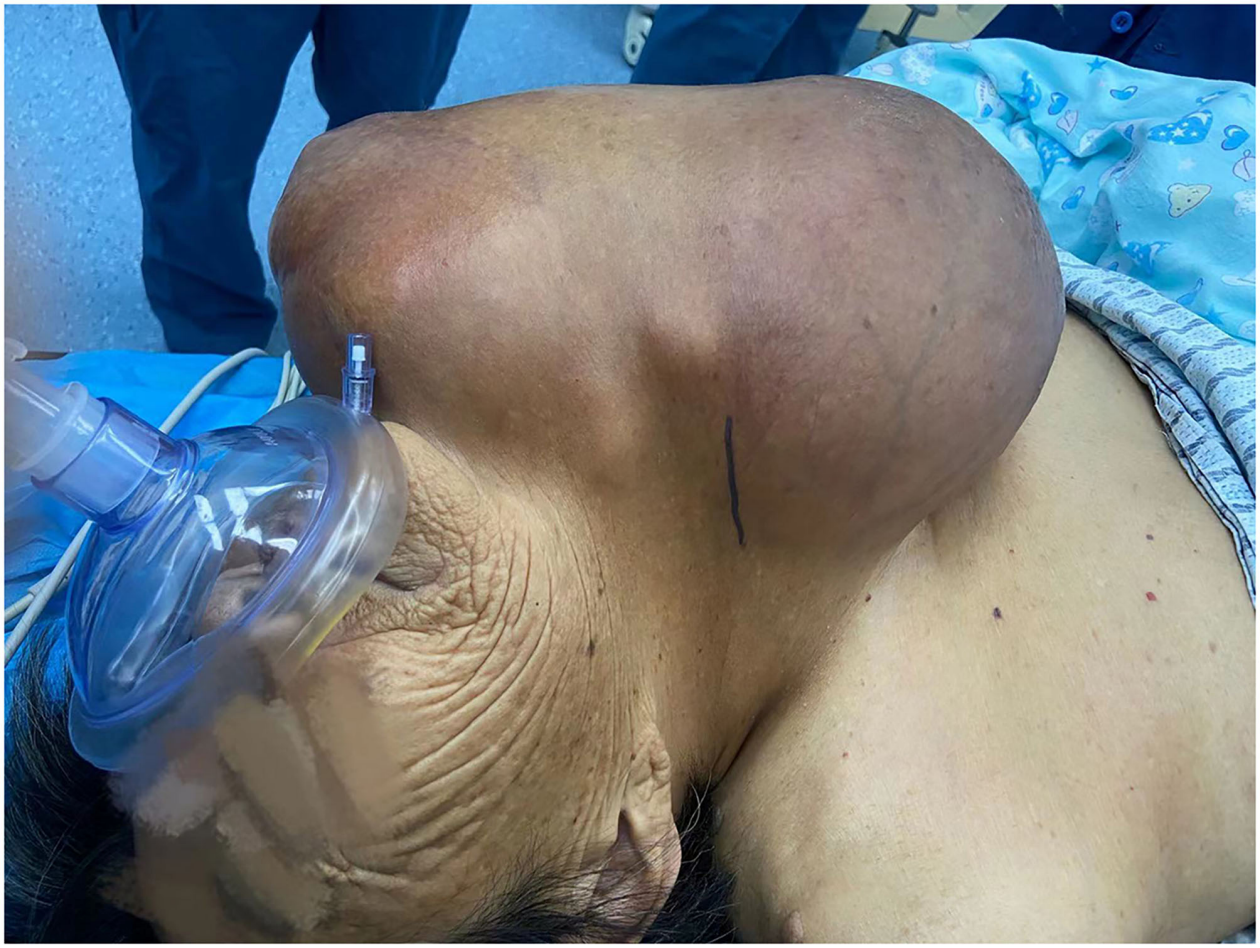
A giant mass in the left anterior neck.

Physical examination showed that there was a 32 × 28 × 25 cm irregular solid mass in the left anterior neck, with a small portion crossing the midline (Figure 1). At palpation the mass was moderately hard and painful, but no vascular murmur was heard. Palpation of the trachea in the suprasternal fossa showed that the trachea deviated to the right.

### Laboratory Examinations

Laboratory examinations included general blood tests, liver function tests, renal function tests, electrolyte tests, thyroid function tests (T3, T4, FT3, FT4, and TSH), parathyroid hormone tests, and tumor markers (AFP, CEA, CA 153, CA 125, and CA 19-9). Except for mild anemia (HGB 94 g/L, normal range 115~150 g/L) and a slight C-reactive protein elevation (21.88 mg/L, normal range 0~10 mg/L), all results were within the normal range.

### Imaging Examination

**Figure 2:**
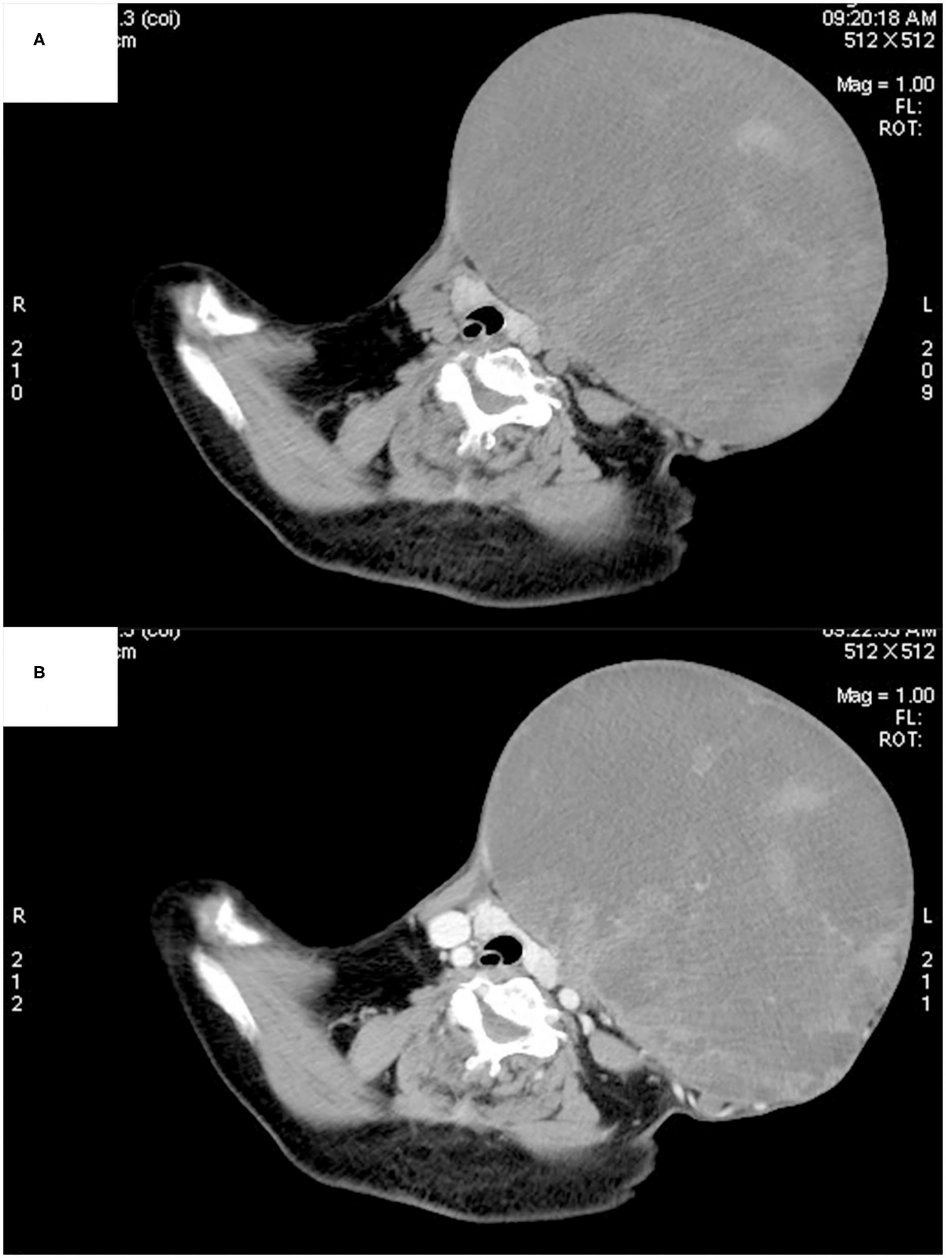
Computed tomography showed a large mixed-density lesion in the left anterior neck, with the trachea and thyroid gland being squeezed to the right side. The internal density of the mass was uneven, with patchy high-density shadows **(A)**. No obvious enhancement was found in the arterial phase **(B)**.

Ultrasonography showed a large mixed echogenic mass with a clear boundary and irregular shape in the middle of the neck. The trachea and thyroid gland were squeezed to the right side. The thyroid had normal morphology, and the capsule was normal. Several heterogeneous hypoechoic and mixed echoes were found in the left level I and level II regions of the neck. Computed tomography also showed a large mixed-density lesion in the left anterior neck, with a maximum cross-sectional area of ~22 × 16 cm. The internal density of the mass was uneven, with patchy high-density shadows. The CT value of the solid area was ~55 Hounsfield units (HU) and that of the cystic area was ~20 HU (Figure 2A). After contrast infusion, there was no obvious enhancement of the mass (Figure 2B). Several small nodular lesions with radiological features similar to those of the large mass were seen in the left parapharyngeal space. No obvious enlarged lymph nodes were found nearby. The trachea was found moderately deviated to the right side, but there was no tracheal collapse.

### Ultrasound-Guided Core Needle Biopsy

**Figure 3:**
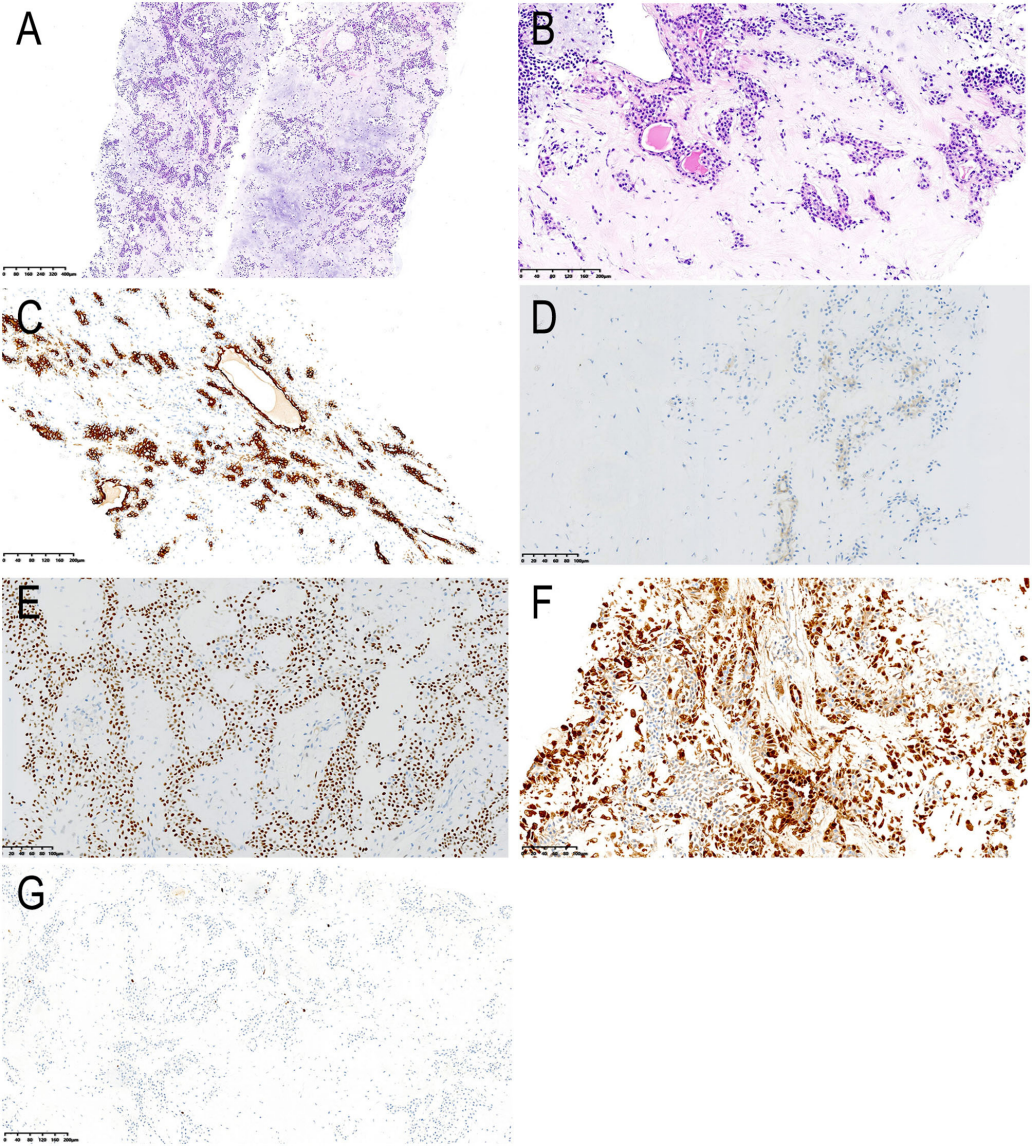
Histological and immunohistochemical features. **(A,B)** Haematoxylin-eosin (HE) staining showing ductal cells, myoepithelial cells and prominent chondromyxoid matrix background. The cuboidal ductal epithelium forms a glandular tubular structure, with red secretion in the lumen; the myoepithelial cells surround the glandular tubular structure or are scattered across the chondromyxoid matrix; the cellular atypia is not obvious (magnification, A × 50, B × 200). By immunohistochemistry, the ductal cells are strongly positive for CK7 **(C)** and positive for CD117 **(D)**, while myoepithelial cells are positive for p63 **(E)** and S-100 **(F)**. Less than 1 % cells were positive for Ki-67 **(G)**. The final diagnosis was pleomorphic adenoma [magnification, **(C–F)** × 200, G × 100].

We arranged an ultrasound-guided percutaneous core needle biopsy. Haematoxylin-eosin (HE) staining showed ductal cells, myoepithelial cells and prominent chondromyxoid matrix background (Figures 3A,B). Immunohistochemically, ductal cells were strongly positive for CK7 and positive for CD117, while myoepithelial cells are positive for p63 and S-100 (Figures 3C–F). The proliferative index, assessed by Ki-67, was <1% positive (Figure 3G). The pathologist suspected a diagnosis of a pleomorphic adenoma and suggested further surgical resection to determine whether malignant transformation had occurred.

### Treatment

The patient underwent surgical excision of the mass after discussion with a multidisciplinary team. For the potential risks in managing the airway, the patient received awake fiber optic transoral intubation. Intraoperative findings showed that the tumor had a complete capsule and did not involve the skin. The tumor originated from the left submandibular fossa. Since the flap was redundant, we removed the tumor and some normal skin attached to the tumor surface. After complete resection of the tumor and its capsule, we found the left submandibular gland atrophied, which may be due to the long-term compression of the tumor. Then we resected the left atrophied submandibular gland. After that, we pulled the anterior and posterior bellies of the left digastric muscle to each side, respectively, to completely expose the lower parapharyngeal space. Then, small tumors located in parapharyngeal space were removed without mandibulotomy and damage to the surrounding important nerves and vessels. Due to the benign nature of PAs, we did not perform a neck dissection. Surgery was uneventful; for safe airway management, the patient was transferred to the intensive care unit for delayed extubation.

### Final Pathology

**Figure 4:**
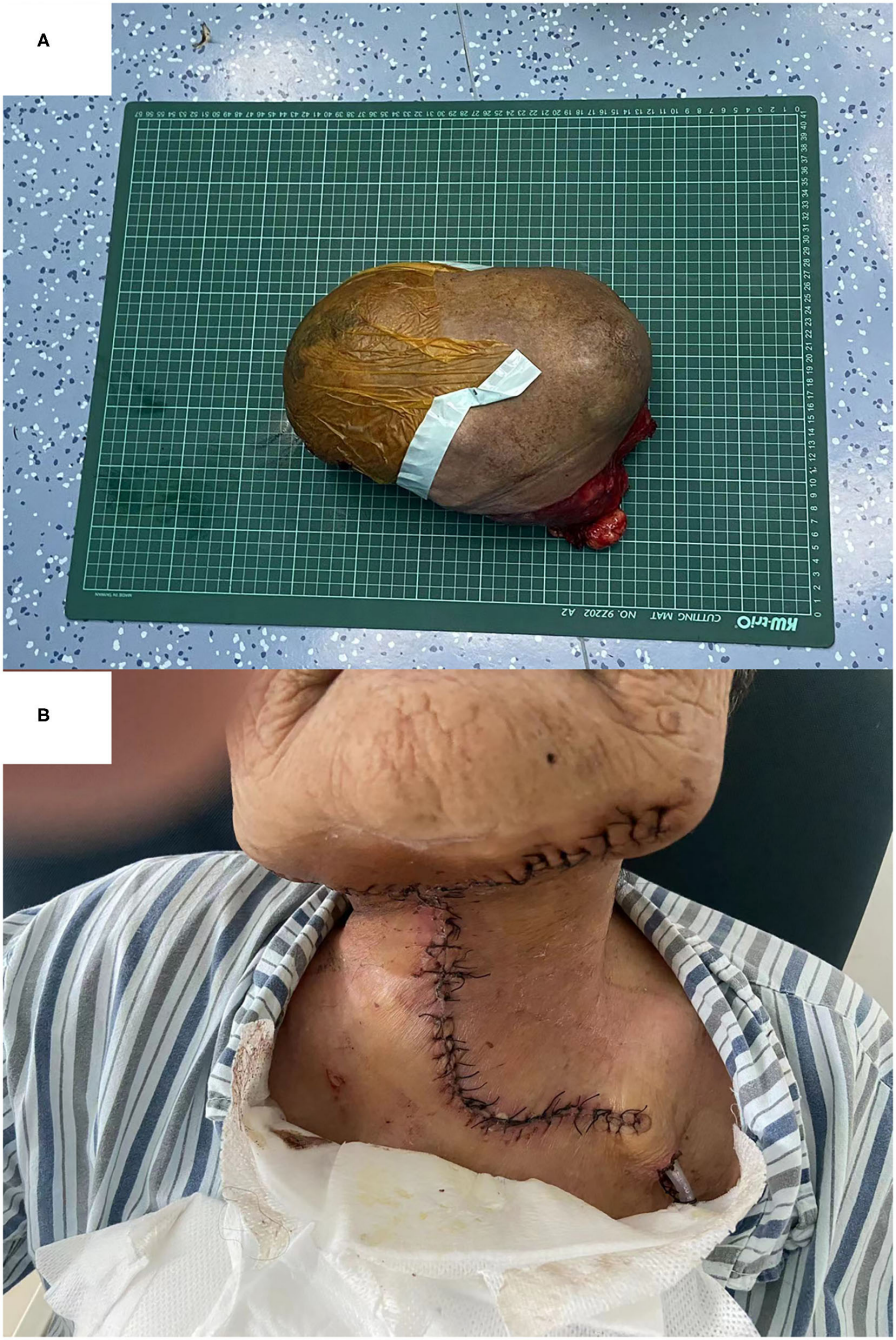
Gross view of the resected specimen **(A)** and appearance of the incision on the neck **(B)**.

On gross examination, the tumor was well-circumscribed with a fibrous capsule. It weighed 5.2 kg, and the dimensions were 32 × 28 × 25 cm (Figure 4A). The cut face was grayish yellow or white, with cystic degeneration and necrosis in focal areas. In the fat surrounding the tumor, there were multiple satellite nodules measuring 1–5 cm. The results of H&E staining and immunohistochemistry were the same as those of the core needle biopsy. A PA of the submandibular gland was diagnosed as a result of these findings.

### Outcome and Follow-Up

The patient recovered well after surgery (Figure 4B). All of the above histopathological findings were congruent with a completely excised PA with a high risk of recurrence. The patient received no further treatment due to her age and was closely followed up.

## Discussion

PAs, also known as “benign mixed tumors,” were first described by Billroth in 1859 and are the most common salivary gland neoplasms to date (3). Although PAs can exhibit striking morphological diversity, they are not real “mixed tumors” derived from more than one germ layer but are of purely epithelial origin, with ductal and pluripotent myoepithelial cells. PAs have an annual incidence rate of ~2.4–3.05 per 100,000 people worldwide (4). Approximately 80–90% of PAs occur in the parotid gland, and only ~5% occur in the submandibular gland (5). PAs most commonly manifest as non-tender, slowly growing nodular lesions in the salivary gland area. The size of PAs usually varies from 2 to 6 cm in the main salivary glands (6). Sometimes, PAs can grow into giant nodules (7–9). In our patient, the tumor grew from the submandibular gland to the anterior neck along with the subcutaneous loose tissue and had become a large tumor. To the best of our knowledge, this is the largest PA of the submandibular gland reported to date.

The pathogenesis of PAs is still not clear. According to some researchers, the pathogenesis of PAs is related to oncogenic simian virus infection, while others believe it is related to exposure to radiation (10). Because PAs grow slowly over years or decades and have diverse well-differentiated components, PAs are generally considered to be benign tumors.

Ultrasonography (US)-guided fine needle aspiration cytology and core needle biopsy are safe and quick methods for the preoperative diagnosis of superficial masses. In one study with 212 patients with PAs, the sensitivity and specificity of the cytological diagnosis were 92.6 and 98.4%, respectively (11). The diversity of the morphological features of PAs is the main cause of misdiagnosis; therefore, cytopathologists need to be familiar with these characteristics (11, 12). Computed tomography (CT) and magnetic resonance imaging (MRI) are commonly used imaging modalities to determine the spatial location of a mass in the neck, its impact on adjacent structures and the status of surrounding lymph nodes. Certain CT features, such as lobulation, homogeneity, and delayed enhancement, can suggest the diagnosis of a PA, but these findings are not specific to the tumor. It should be noted that the CT features of PAs are also affected by the different proportions of internal components. In one report with 262 PAs, CT with high contrast enhancement was correlated with a high proportion of epithelial components in histopathological findings (13). MRI usually shows pleomorphic adenomas as well-circumscribed, homogeneous masses with low intensity on T1-weighted images and high intensity on T2-weighted images. Zaghi et al. identified “high probability” MRI criteria for PA diagnosis, with 95.1% specificity and 43.9% sensitivity (14). Other studies have also shown that MRI may be used alone (without FNAB) for a subset of PAs to narrow the possibilities of differential diagnosis (15, 16).

Surgical removal of the nodule and the affected gland is thought to be the standard treatment (17). However, complete excision of the whole affected gland is mainly performed in the submandibular PA, but rarely on the parotid PA. For parotid PA, many surgeons prefer partial gland resection to preserve the facial nerve (18). Our patient had a history of an excision of a neck mass 40 years prior to presentation at another hospital. Although relevant information could not be found, based on our imaging findings and surgical findings, we speculate that the neck mass 40 years prior was also a PA. Thus, our case was interpreted as a recurrent PA. When a PA recurs, surgical resection followed by radiotherapy is thought to be the best treatment because salvage surgery is not as likely to be curative as primary surgery (19). At present, there is no established chemotherapy regimen for the treatment of PAs or recurrent PAs. In fact, radiation therapy for the treatment of recurrent PA remains controversial because, on the one hand, it complicates possible corrective surgery, and on the other hand, the risk of developing secondary radiation neoplasms is not zero, especially in young patients (20). While several retrospective studies have reported improved local control with radiotherapy in recurrent PA (21, 22). A systematic review shows that adjuvant radiotherapy is of benefit in reducing the recurrence rate of recurrent PAs in those with adverse prognosis factors such as multinodular recurrence (23). However, due to the lack of prospective evidence, it is recommended to be used cautiously in patients at high risk for further recurrence. In our patient, postoperative pathology revealed that there were multiple satellite nodules measuring 1–5 cm in the surrounding fat, which means that the patient still has a high risk of recurrence. Considering the patient's age, however, she did not receive radiotherapy.

What should not be neglected is that in 5–10% of patients, a pleomorphic adenoma can transform into a carcinoma (known as carcinoma ex pleomorphic adenoma), which most often develops in the parotid gland and represents 2–3% of all parotid neoplasms (3, 24). This malignant transformation is more common in recurrent pleomorphic adenomas. The clinical manifestations are mostly painless progressive masses with sudden rapid growth in a short period. If the tumor infiltrates the nerves and surrounding tissues, there will be symptoms such as pain, numbness, facial paralysis and skin ulcers. The longer the pleomorphic adenoma exists, the higher the risk of malignant transformation (25).

## Conclusion

We report a giant PA of the submandibular gland, most likely a recurrent PA. The lesion protruded to the anterior area of the neck, mimicking a tumor of thyroid origin. Preoperative core needle biopsy provided an accurate diagnosis. CT facilitated the resectability assessment. Postoperative pathology revealed satellite foci in the tumor-adjacent adipose tissue. PAs are slow-growing benign tumors, but they easily recur and may transform into malignancies. Clinicians, pathologists and imaging specialists should pay attention to these tumors.

## Data Availability Statement

The raw data supporting the conclusions of this article will be made available by the authors, without undue reservation.

## Ethics Statement

Ethical review and approval was not required for the study on human participants in accordance with the local legislation and institutional requirements. Written informed consent was obtained from the participants legal guardian/next of kin, for the publication of any potentially identifiable images or data included in this article.

## Author Contributions

ZW, DL, TG, and AX: conceived the study. DL, SP, HW, and YL: collected the data. ZW, DL, and TG: analyzed the data. YW, XZ, and LL: provided guidance on data analysis. ZW: wrote the paper. TG and AX: revised the paper. All authors contributed to the article and approved the submitted version.

## Funding

This study was supported by grants from the Basic and Clinical Cooperative Research and Promotion Program of Anhui Medical University (2020xkjT045) and the National and Provincial Key Specialty Construction Plan (No. Z155080000004).

## Conflict of Interest

The authors declare that the research was conducted in the absence of any commercial or financial relationships that could be construed as a potential conflict of interest.

## Publisher's Note

All claims expressed in this article are solely those of the authors and do not necessarily represent those of their affiliated organizations, or those of the publisher, the editors and the reviewers. Any product that may be evaluated in this article, or claim that may be made by its manufacturer, is not guaranteed or endorsed by the publisher.
